# Research Progress of Natural Matrine Compounds and Synthetic Matrine Derivatives

**DOI:** 10.3390/molecules28155780

**Published:** 2023-07-31

**Authors:** Jinlei Li, Shijie Wei, Davies Marabada, Zhizhong Wang, Qing Huang

**Affiliations:** 1 School of Pharmacy, Ningxia Medical University, Yinchuan 750004, China; lijinlei0025@163.com (J.L.); marabadadavies@gmail.com (D.M.); 2 Pharmacy Department, General Hospital of Ningxia Medical University, Yinchuan 750004, China; nxwsj1978@163.com

**Keywords:** matrine, *Sophora flavescens*, structural modification, matrine derivatives, matrine-type alkaloids, biological activity

## Abstract

Matrine is a quinoline alkaloid extracted and separated from the dried root, fruit, and other parts of the plant *Sophora flavescens* using an organic solvent. Matrine exhibits a variety of biological activities and is widely used in pharmacy, agronomy, and other fields. Due to its low bioavailability, poor chemical stability, and toxicity to the central nervous system, a large number of researchers have searched for matrine derivatives with higher biological activity and safety by modifying its structure. In this review article, the research progress of matrine derivatives obtained using two methods (extraction from *Sophora flavescens* and structural modifications) from 2018 to 2022 in terms of pharmacological activity, mechanism of action, and structure–activity relationship are presented. The modification of matrine over the past five years has been mainly on the D-ring. Many new matrine alkaloids have been extracted from natural products, some of which have good pharmacological activity, which broadens the strategy for matrine structural modification in the future.

## 1. Introduction

**Figure 1:**
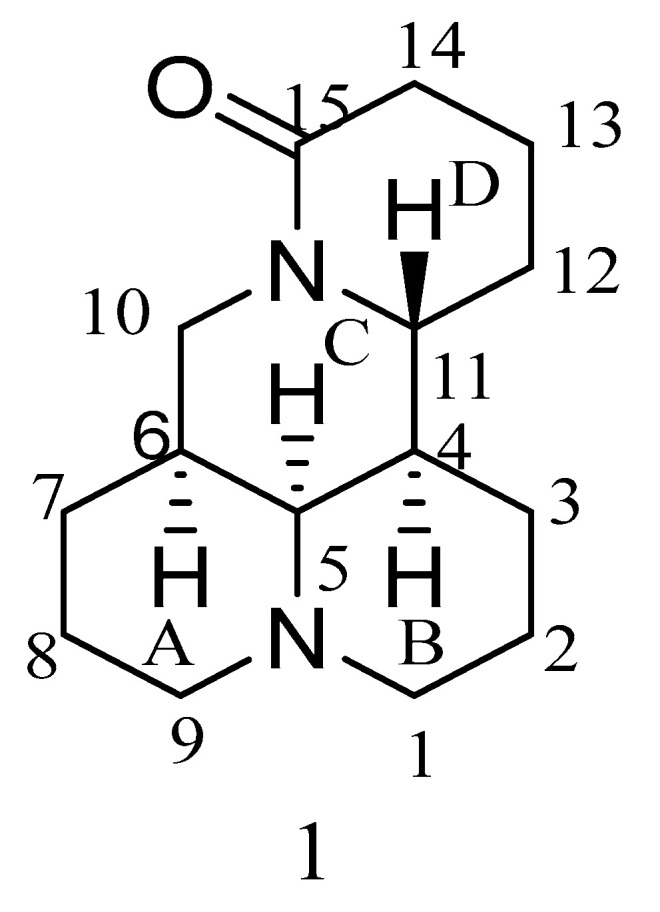
Structure of matrine.

Matrine (compound **1** in Figure 1) is a quinoline alkaloid extracted and separated from the dried root, fruit, and other parts of the legume species *Sophora flavescens* using an organic solvent. Matrine has a variety of pharmacological activities, such as anti-tumor, antiviral, anti-inflammatory, liver protection, anti-arrhythmia, antibacterial, antipyretic, analgesic, and so on [1,2,3,4,5,6,7,8,9,10,11,12,13,14,15,16,17,18,19,20,21,22,23,24]. It is mainly used in the treatment of chronic hepatitis, allergic dermatitis, gynecological diseases, and cardiovascular diseases in the clinic. Because of the extensive and excellent biological and pharmaceutical properties of matrine, many researchers have carried out a lot of research. The study of the anti-tumor activity of matrine has become one of the research hotspots in recent years.

However, as a natural product, matrine has some problems, such as a relatively weak activity and severe toxicity. Matrine has good pharmacological activity but low lipid solubility, poor bioavailability, and a short half-life in vivo [25]. A pharmacokinetic study of matrine in rats showed that the bioavailability of matrine in rats was only 18.5% [26]. A large number of experimental results showed that modifications to the D-ring can improve these deficiencies of matrine. In evaluating the efficacy of a drug, toxicity and safety should be primarily taken into account. The hepatotoxicity, developmental toxicity, and neurotoxicity of matrine are the most commonly reported [27,28,29], and to date, few studies have been reported to reduce the toxicity of matrine. Studies have shown that both oxymatrine and matrine have certain toxicities in mice, but the toxicity of oxymatrine is significantly weaker than that of matrine. It is speculated that the toxicity difference may be related to the oxidation of the tertiary amine structure on the matrine ring to a quaternary amine [30]. Therefore, further medicinal and chemical modifications become necessary. At present, a large number of studies have reported that matrine and its derivatives have good biological activities as drugs and pesticides [31,32,33,34,35,36,37,38,39,40,41,42].

The research of matrine derivatives in recent years mainly includes the following two aspects. First, with technological progress in separation and purification, structure identification, bioactivity-oriented tracking, and other aspects, the extraction and separation of natural compounds from *Sophora flavescens* and the analysis of its components have been extensively studied in order to find novel alkaloids with higher activities. With the increasing investment in the development of new drugs, many new natural matrine-type alkaloids have been discovered and their biological activities have been tested. These newly discovered structures may provide new ideas for further study of the structural modifications of matrine. The second aspect is to modify the structure of matrine using chemical methods, and then design synthetic routes and evaluate the biological activities of the new compounds. Researchers have performed a lot of structural modifications on matrine. Published studies mainly focused on the modification of the D-ring, the derivatization of C-13, C-14 and C-15, opening the D-ring, and combining the D-ring with some aromatic rings. The structure–activity relationship of matrine has also been proposed in many studies on structural modifications.

In order to develop new drugs with high activity and low toxicity more accurately, a thorough and comprehensive grasp of previous research is essential. In this overview, the most critical research of matrine derivatives in the past five years from the above two aspects is summarized.

## 2. Novel Matrine-Type Alkaloids

**Figure 2:**
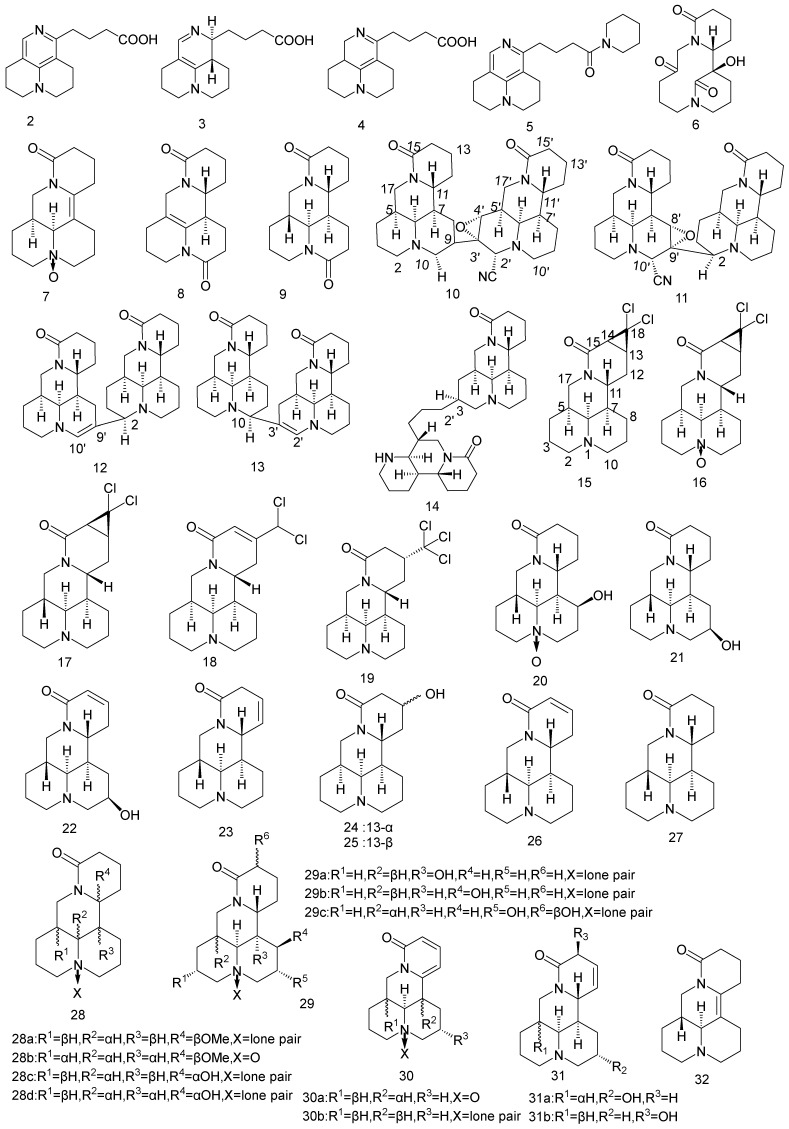

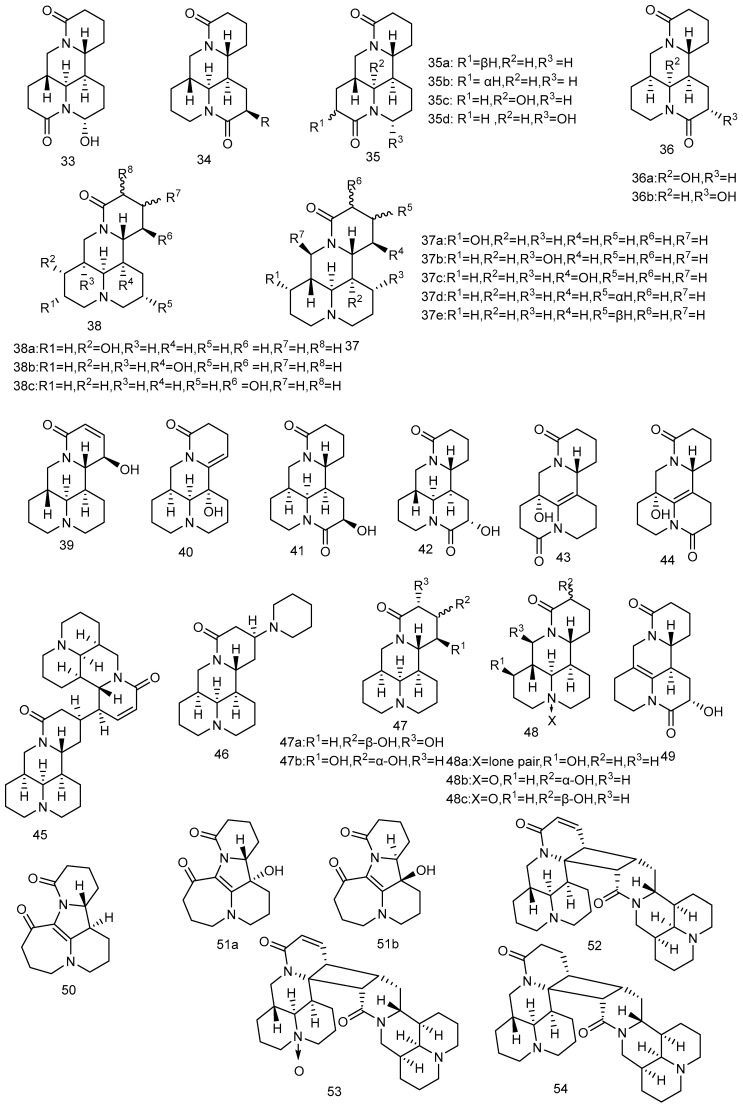
Chemical structure of newly discovered matrine alkaloids reported from 2018 to 2022 [43,44,45,46,47,48,49,50,51,52,53]. Adapted with permission from J. Nat. Prod. 2018, 81, 2259–2265. Copyright 2018, American Chemical Society; Adapted with permission from J. Nat. Prod. 2019, 82, 3227–3232. Copyright 2018, American Chemical Society; Copyright 2021 Luo, Tu, Yin, Fan, Chen, Wu, Ding, Li, Wang and Zhang.

In this part, 74 new matrine alkaloids extracted and isolated from matrine reported from 2018 to 2023 are summarized. All the chemical structures are listed in Figure 2 and the reported activity studies of these new compounds are summarized in Table 1. The structure–activity relationship of these compounds is preliminarily analyzed in the following paragraphs.

In 2018, Zhang et al. [43] reported compounds **2**–**9** were isolated from the root of *Sophora flavescens*. Compounds **2**–**4** are the first natural matrine-type alkaloids with open D-rings. Compound **5** represents an unprecedented dimeric model constructed from matrine and piperidine; compound **6** is the first matrine-type alkaloid with a C-5-C-6 bond cleavage. Compounds **2**, **5** and **6** showed anti-HBV activity comparable to matrine. Comparing the activity and structure of compounds **2** and **5**, it is possible that the introduction of the pyridine ring on C-15 after ring-opening can increase the anti-HBV activity. The anti-HBV activity of compound **6** suggests that opening the C-5-C-6 bond may have a positive effect on enhancing activity.

Fan et al. [44] reported that five new matrine-type alkaloid dimers (compounds **10**–**14**) were isolated from the stems of *Sophora alopecuroides*. Compounds **10** and **11** are the first dimeric matrine-type alkaloids with one cyano group and one epoxy group, while compounds **12** and **13** are dimeric matrine-type alkaloids linked by C-2-C-9′ and C-10-C-3′ bonds. The anti-inflammatory activities of these five compounds were evaluated. As shown in Table 1, compounds **10**–**13** inhibit the production of the inflammatory factor IL-6 (interleukin-6), whereas compound **14** does not. It was suggested that the two matrine structures may act together. Compounds **11**–**14** inhibit the release of TNF-α (tumor necrosis factor-α), while 10 does not, indicating that the oxidation of C-3 and C-4 may decrease drug activity. Compounds **11** and **12** show higher activity than the other compounds, implying the C-2-C-9′ bond may be beneficial to enhance the anti-inflammatory activity. Moreover, compound **11** shows the strongest anti-inflammatory activity (stronger than that of matrine), suggesting oxidation of C-8 and C-9 may favor activity. Furthermore, compounds **12** and **13** exhibit similar activities to matrine but no apparent consistency in their inhibitory effects on TNF-α and IL-6, suggesting that the compounds may have different effects on the upstream regulators of these cytokines.

In 2020, Wang et al. [45] discovered five new matrine alkaloids (Figure 2) from *Sophora alopecuroides*. The structures of compounds **15** to **19** are fused with dichlorocyclopropane on the D-ring of matrine, and the structures of compounds **18** and **19** are added with dichloromethyl on the D-ring. The results of immunosuppression experiments with cyclosporine as a positive control showed that compound **18** has a strong inhibitory activity. In the structure–activity analysis of the compounds, it is assumed that dichlorocyclopropane fused to the D ring and trichloromethyl bonded to C-13 had a negative effect on the activity, which is probably due to high steric hindrance

Compounds **20**–**22** are hydroxymatrine, reported in 2021, whose hydroxyl group is attached to the 8 or 9 positions of the B ring [46]. The experimental results of the inhibitory activity of the three compounds on the production of NO induced by LPS in RAW 264.7 cells show that compound **20** has a significant inhibitory effect. The structure–activity analysis showed that oxidation at the N-1 and β-OH at C-8 was crucial for its anti-inflammatory activity.

Five novel matrine-type alkaloids (Figure 2) were isolated from the roots of *Sophora tonkinesis* including two novel compounds (compounds **23** and **26**) and a novel natural product (compound **24**) [47]. The inhibitory effects of compounds **23**–**27** on LPS-induced production of inflammatory factors TNF-α and IL-6 by RAW 264.7 macrophages were investigated, among which compound **23** has the strongest anti-inflammatory activity (stronger than that of matrine).

In 2021 [48], 12 matrine alkaloids (compounds **28a**–**28d**, **29a**–**29c**, **30a**–**30b**, **31a**–**31b**, and **32**) isolated from *Sophora alopecuroides* were evaluated for their inhibitory effects on LPS-induced NO production by RAW 264.7 cells. All compounds showed anti-inflammatory activity, with compound **31b** showing stronger activity than matrine. Further studies on the new compound **31b** show that it also inhibits the expression levels of iNOS (inducible isoform of nitric oxide synthase) and COX-2 (cyclooxygenase-2), indicating that compound **31b** has the potential to be developed into an anti-inflammatory drug.

In a phytochemistry study of the water-soluble alkaloids from *Sophora alopecuroides*, Luo et al. [49] discovered 31 new matrine alkaloids (compounds **33**–**49**), covering almost all the oxygen substitutions in the matrine-type structure. Compounds **36**–**38** are rare bisamidomatrine alkaloids. Compounds **33**–**40** were evaluated for their anti-neuroinflammatory activities in LPS-stimulated BV2 microglia. All matrine derivatives show inhibitory effects on neuroinflammation. The discovery of compounds **33**–**40** provide new methods for developing novel modification strategies based on A-ring or B-ring.

The inhibitory effects of compounds **41**–**44** on TNF-α and IL-6 in LPS-stimulated RAW264.7 cells were evaluated [50]. The results showed (Table 1) that compounds **43** and **44** have obvious anti-inflammatory effects in a dose-dependent manner. The structure–activity relationship analysis suggested that the double bonds of C-6 and C-7 could promote anti-inflammatory activity.

Compound **45** is the first dimerized matrine type alkaloid linked by C-13 and 12′. The cytotoxicity against four cancer cells and anti-inflammatory activity of compounds **45**–**49** were evaluated [51]. The results showed that the new compounds have anti-inflammatory activity without significant cytotoxicity. Compared with the activity of the previously discovered dimer matrine-type alkaloids, compound **45** showed superior activity, suggesting that the dimer linked by C-13 and C-12′ bonds has a positive effect on anti-inflammatory activity.

Compounds **50**, **51a,** and **51b** have an unprecedented rearrangement fusing the 7/6/5/6 tetracyclic skeleton with a diazocyclic heptane structure [52]. Compound **51** is a pair of enantiomers with a 95:5 ratio of **51a** to **51b**. The MTT(3-(4,5)-dimethylthiahiazo(-z-y1)-3,5-di-phenytetrazoliumromide) assay was used to detect anti-proliferation activities of compounds **50** and **51** on A549 cells. Compound **50** had a strong anti-proliferation activity on A549 cells with an IC_50_ value of 7.58 ± 2.47 μM at 72 h and induced cell cycle arrest in G1 phase in a dose-dependent manner. The results showed that compound **50** has the potential to be developed into a new anti-lung cancer drug.

Compounds **52**–**54** are three new [2 + 2] cycloaddition alkaloid dimers isolated and identified from *Sophora flavescens*, with unprecedented 6/6/6/6/4/6/6/6/6 nonacyclic skeleton, containing 11 chiral centers [53]. The results of in vitro experiments showed that compound **52** could inhibit APAP (acetaminophen)-induced apoptosis and ROS (reactive oxygen species) levels in HepG2 cells alleviating the damage of APAP on hepatocytes, indicating that compound **52** has a good liver protective activity.

## 3. Modification on the D-Ring

### 3.1. Modification at Position C-13

C-13 is one of the most common modification sites for enhancing the pharmacological activity of matrine. In the past five years, crucial studies on modification at this site are summarized in this part, including the synthetic route of derivatives, pharmacological activity studies (Table 2), pharmacological mechanisms, and structure–activity relationship analysis.

**Scheme 1:**
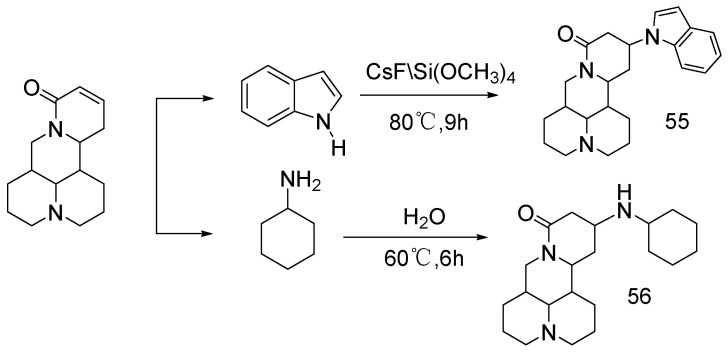
The synthetic route of compounds **55** and **56** [54].

Cheng et al. [54] reported the synthesis of compounds **55** and **56** (Scheme 1) using sophocarpine as the raw material, introducing indole and cyclohexylamine at C-13. The results of bioassay in vitro showed that the cytotoxicity of these two compounds to Hela229 was greater than that of matrine. As reported in previous studies, the introduction of indole or cyclohexylamine groups at D-ring 13 of matrine skeleton can greatly improve its proliferative activity against cancer cells. It was noted that compound **55** showed stronger activity than **56**, possibly increasing its permeability to the cell membrane due to the introduction of indole.

**Scheme 2:**
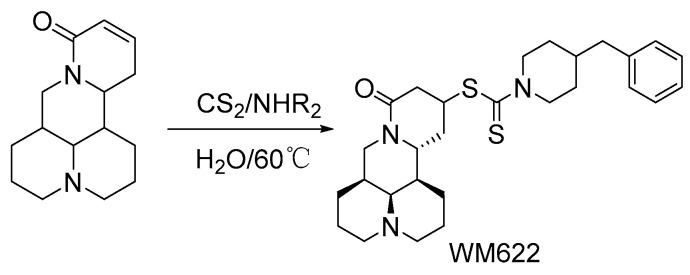
The synthetic route of WM622 [55].

When matrine was previously used in anti-hepatoma studies, its pharmacological activity was found to be limited. Sun et al. [55] reported a series of matrine derivatives that were obtained by chemical structure modifications and found that WM622 had high lipid solubility and antiproliferation activity in HCC cells. The synthesis of WM622 (Scheme 2) mechanism includes affinity addition and Michael addition. The molecular mechanism of WM622 affecting the biological behavior of HCC cells was further studied. The results showed that WM622 could inhibit the proliferation, invasion, and migration of HCC cells. It can induce apoptosis and cell cycle arrest in HCC cells in a concentration-dependent manner and inhibit tumor growth probably by inhibiting the PI3K (Phosphatidylinositol3-kinase)/AKT/GSK3β (recombinant Glycogen Synthase Kinase 3 Beta) pathway. Moreover, WM622 is more effective than 5-fluorouracil in inhibiting tumor growth in tumor-bearing mice.

**Scheme 3:**
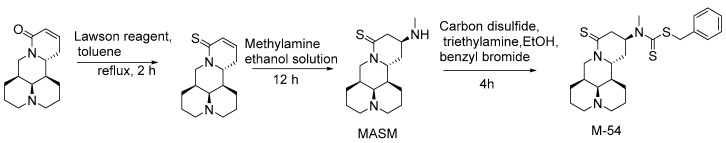
The synthetic route of M-54 [60].

**Scheme 4:**
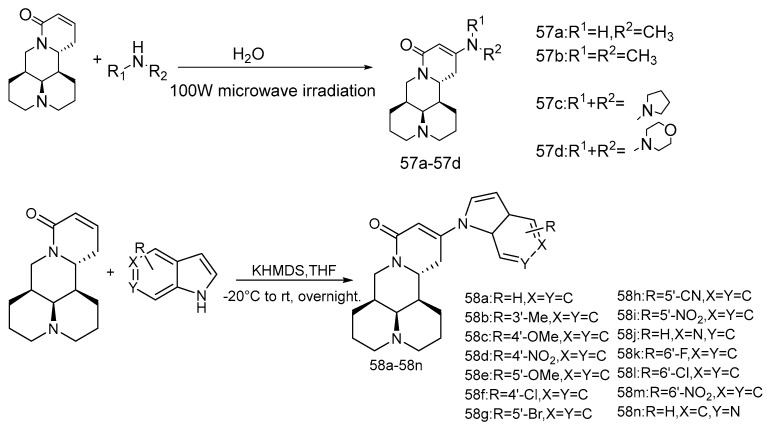
The synthetic route of compounds **57a**–**57d** and **58a**–**58n** [61].

MASM, also known as M19 (compound **56**), was first synthesized by Hu et al. [56] of the Naval Medical University. In 2019, Zou et al. [57] reported the cytotoxicity and mechanism of action of MASM on four cancer cells (A549, MCF-7, MDA-MB-231 and Hela). The mechanism of MASM-induced apoptosis was studied with flow cytometry, Western blot and immunofluorescence. It is suggested that the effect of MASM on cancer cells is regulated by ROS production. Xu et al. [58] reported that RPS5 (ribosomal protein S5) is the direct target of MASM on anti-liver fibrosis in 2014. Studies have shown [59] that MASM has anti-osteoporosis activity, which also acts on RPS5. In another study [60], in view of the characteristics of the unstable physical and chemical properties of MASM, in vitro the hydrophobic groups were introduced into the MASM, and then the optimal and achievable target compound M54 was selected by computer simulation docking software. The synthesis route of M54 is shown in Scheme 3. The results from in vitro and in vivo experiments showed that compared with MASM, the stability of M54 is significantly improved, the inhibitory effect on osteoclast differentiation and maturation is enhanced, and the cytotoxicity is significantly reduced. Western blot analysis showed that M54 inhibits RANKL-induced osteoclastogenesis. RANKL-RANK signaling induces NFATc1 expression through NF-κB, MAPKs, and PI3K/AKT pathways. M54 acts on the RPS5 and blocks NF-κB, MAPKs, and PI3K/AKT pathways and, subsequently, suppresses NFATc1 expression. Based on the anti-hepatic fiber activity of MASM and the fact that indole is an antifibrotic drug with a unique structure, Li et al. [61] reported the synthesis (Scheme 4) of a series of matrine derivatives (compound **57a**–**57d** and **58a**–**58n**) obtained by substituting indole groups for aminomethyl of MASM. The inhibitory effect of all new compounds on TGF-β1 (Transforming growth factor beta 1)-induced total collagen accumulation in the lung fibroblast MRC-5 cell line was also evaluated. A series of experimental data suggest 58f may be a potential drug for the treatment of idiopathic pulmonary fibrosis by inhibiting the TGFβ/Smad signaling pathway.

**Scheme 5:**
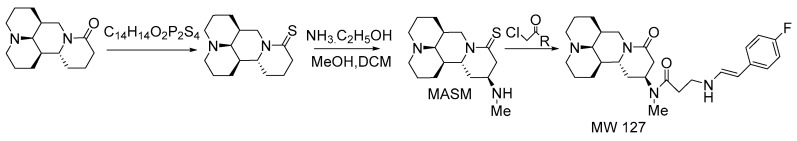
The synthetic route of MW127 [63].

Survivin is also known as a baculoviral inhibitor of the apoptosis repeat containing protein 5 (BIRC5). A large number of clinical samples have shown that Survivin is overexpressed in almost all types of malignant tumors [62]. Studies have shown that matrine derivatives have Survivin inhibitory properties, good anti-tumor effects, and few side effects. Yin et al. [63] established a Survivin-targeted drug screening platform based on the expression mechanism of Survivin. Using this platform, the matrine derivative WM-127 with the best inhibitory effect on liver cancer tumor growth was selected, and the synthesis route (Scheme 5) was followed by Michael addition via MASM to generate the final product. Western blot experiments confirmed the consistent expression levels of Survivin and EGFP (enhanced green fluorescent protein) in the cell model HepG2-Sur5P-EGFP-Sur3U. It was observed by fluorescence microscopy that EGFP expression was significantly inhibited after WM127 was applied to cell models HepG2-Sur5P-EGFP-Sur3U, and WM127 is considered to be a powerful “Survivin inhibitor”. The effects of MW127 on Survivin expression, cell proliferation and apoptosis in HCC cells were studied, which further confirmed that MW127 is a promising drug for HCC treatment. Mechanistic studies showed that WM-127 suppressed the activity of the Survivin/b-catenin pathway and the expression of Bax to induce cell cycle arrest and apoptosis.

**Table 2:** Studies on the activity of matrine derivatives structurally modified on matrine C-13 from 2018 to 2022.

Compound	Activity Test	Method	Result	References
**55–56**	Antiproliferative activities assays against the human cancer cell lines Hela229	MTT assay	The IC_50_ values of sophorine, matrine, compounds **55** and **56** against Hela229 cells at 72 h were 1.50 ± 0.80, 1.53 ± 0.32, 0.42 ± 0.12 and 0.12 ± 0.03 mM, respectively	[54]
**WM622**	Antiproliferative activity against hepatocellular carcinoma cells	MTT assay	The IC_50_ of WM622 was 34 µM for LM3, and 41 µM for Hep3B, respectively. The IC_50_ of matrine was 801 µM for LM3, and 845 µM for Hep3B, respectively.	[55]
	Inhibition on migration of HCC cells	Transwell chamber assay	WM622 can inhibit the migration activity of HCC cells and the effect was better than matrine	[55]
	Inducing apoptosis of HCC cells	Flow cytometry	The apoptosis rate of LM3 cells were 5.68 ± 0.17%, 9.48 ± 0.43%, 12.2 ± 0.30% and 16.43 ± 0.28% in response to treatment with 0, 10, 20 and 30 µM of WM622, respectively. The apoptosis rate of Hep3B cells were 4.70 ± 1.52%, 7.56 ± 1.42%, 9.52 ± 0.98% and 14.20 ± 0.60% in response to treatment with 0, 10, 20 and 30 µM of WM622, respectively	[55]
	Growth inhibition of HCC xenografts in nude mice	Balb/c nude mice	The effect of WM622 on tumor growth was stronger than that of 5-Fu; Western experiments showed that expression levels of p-PI3K (Tyr458), p-AKT(Thr308), p-EGFR(Tyr1068) and p-GSK3β(Ser9) were down-regulated and the level of PTEN was up-regulated dose dependently	[55]
MASM	Effects of MASM on cell viability and cellular toxicity	MTT assay LDH assay	MASM induced a dose- and time-dependent inhibitory effect on the viability of A549, MCF-7, MDA-MB-231 and Hela cells	[57]
	Analysis for apoptosis	Flow cytometry LDH assay	At the same dosage MASM induced more apoptosis in MDA-MB-231 and Hela cells than in A549 and MCF-7; MASM induce apoptosis in a dose-dependent manner through caspase 3-dependent manner (in A549, MDA-MB-213 and Hela) and caspase 3-independent manner (in MCF-7, which is caspase 3 deficient)	[57]
	The expression levels of p-Akt, Akt, p-Erk1/2, Erk1/2, p-p38, and p38 were detected by Western blot	Western Blot	MASM inhibited Akt phosphorylation in all four cell types in a dose dependent manner. MASM promotes the phosphorylation of Erk1/2 and p38.	[57]
	Apoptosis of MDA-MA-231 in the presence of ROS scavher N-acetylcysteine	Western Blot	Western blot analysis showed that the activation of Erk1/2 and p38by MASM and the accumulation of LC3-II were also inhibited by NAC	[57]
M-54	The cell differentiation effect of M-54 on BMMCs and RAW264.7 cells	TRAR staining	The number of osteoclasts in BMMCs and RAW264.7 cells treated with M-54 decreased significantly	[60]
	The effect of M-54 on RPS5 PI3K-AKT, NF-κB, and MAPK expression in RANKL-induced osteoclasts	Western blot	Osteoclasts treated with M-54 could increase the expression of RPS5 in a dose-dependent manner, and significantly inhibit phosphorylation of Akt, P65, IKb, ERK, JNK, and P38.	[60]
	Inhibition of bone loss induced by ovariectomy in mice	OVX mouse Micro-CT Analysis	Histological analysis showed that M54 prevented the loss of bone trabeculae	[60]
**57**–**58**	Inhibition of TGF-β1-Induced Collagen Accumulation	Sircol collagen assay	The IC_50_ values of compounds **57a**–**57d** and **58a**–**58n** and matrine on the total collagen accumulation of TGF-β1-stimulated MRC-5 cells were 65.3 ± 4.2, 92.1 ± 1.9, 255.8 ± 22.1, 138.4 ± 9.8, 28.3 ± 3.9, 39.0 ± 0.6, 44.7 ± 3.6, 4.3 ± 0.4, 68.1 ± 5.5, 3.3 ± 0.3, 6.5 ± 1.2, 72.1 ± 4.0, 14.5 ± 1.0, 16.7 ± 3.5, 23.1 ± 5.3, 27.0 ± 1.4, 19.3 ± 2.2, 69.5 ± 8.2, 878 ± 68 µM, respectively	[61]
**58f**	Inhibition Effects of Expression Levels of extracellular matrix Proteins by **58f**	Immunofluorescence Assay	Compared with the TGF-β1 stimulation group, the **58f** treatment group reduced fibronectin expression levels by 40%	[61]
	Inhibition Effects against Fibroblast-to-Myfibroblast Transition by **58f**	Immunofluorescence Assay	The conversion of fibroblasts into myofibroblasts after treatment with compound **58f** was interrupted, and the expression levels of α-SMA and S100A4 were reduced from 774.9% and 537.7% to 45.8% and 17.7%, respectively	[61]
	Inhibition Effects against Smad-Mediated Signaling by **58f**	Immunofluorescence Assay	**58f** could significantly inhibit the cytoplasm-to-nuclear translocation of Smad2/3.	[61]
	Inhibition Effects against TGF-β1 Induced Migration of MRC-5 Cells by **58f**	Scratch Assay	The wound closure area of TGF-β1-stimulated MRC-5 fibroblasts reached 85% within 24 h, 10 μM compound **58f** treated was only 37.7%, 1000 μM matrine treated was 56.6%.	[61]
WM1 series	Effects of WM1 series on EGFP expression in cell models HepG2-Sur5P-EGFP-Sur3U	Fluorescence microscope observation	WM-127 had the strongest inhibitory effect on ECFP in cell models HepG2-Sur5P-EGFP-Sur3U	[63]
WM127	Anti-proliferative activity of WM127 on HCC cells	MTT assay	The IC_50_ values of WM127 against WRL-68, Huh-7 and HepG2 cells were 105.80 ± 5.4, 60.04 ± 2.47 and 52.59 ± 4.29 μg/mL, respectively, and the proliferation of HepG2 and Huh7 cells was inhibited in a dose—and time-dependent manner	[63]
	Effects of WM127 on cell cycle and apoptosis	Flow cytometry	MW127 induced obvious G2 phase arrest in HepG2 and Huh-7 cells, but not in WRL-68 cells; Compared with the control group, the apoptosis rate of HepG2 and Huh-7 cells treated with WM127 was significantly increased, but the apoptosis rate of WRL-68 cells was not significantly increased	[63]
	Effect of WM127 on Survivin/β-catenin pathway in HCC cells and Bax	Western blot; immunohistochemistry	The expression of b-catenin was down-regulated dose-dependently, being consistent with the expression of Survivin, the expression level of Bax was up-regulated in the WM-127-treated HepG2 and Huh-7 cells	[63]

Note: Data are presented as the mean ± SD of three independent experiments.

### 3.2. Modification at Position C-14

Studies on the pharmacological activities of matrine derivatives obtained by modification on C-14 are summarized in Table 3.

**Scheme 6:**
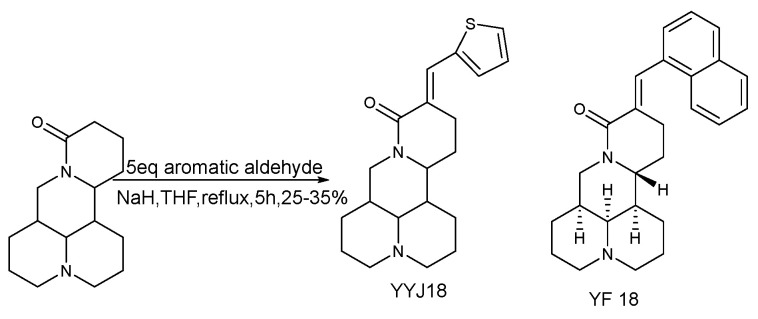
The synthetic route of YYJ18 [66].

The synthesis of YYJ18 was reported as early as 2012, the synthetic route is shown in Scheme 6, and YYJ18 was reported to inhibit the proliferation of nasopharyngeal carcinoma cells in a 2014 report [64,65]. In 2019, Kong [66] reported that YYJ18 can inhibit the proliferation, invasion, and metastasis of nasopharyngeal carcinoma CNE2 and 5–8F cells. Moreover, its mechanisms of action are to regulate the expression of EMT (epithelial-mesenchymal transformation) related genes N-cadherin, E-cadherin, and Vimentin through the miR-146b-5p pathway, and inhibit the phosphorylation of AKT protein, thereby inhibiting the activation of PI3K/AKT signaling pathway.

**Scheme 7:**
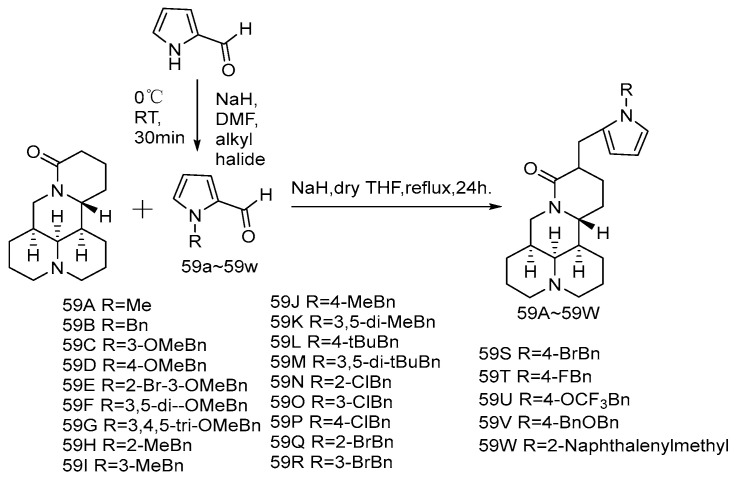
The synthetic route of compounds **59A**–**59W** [68].

**Scheme 8:**
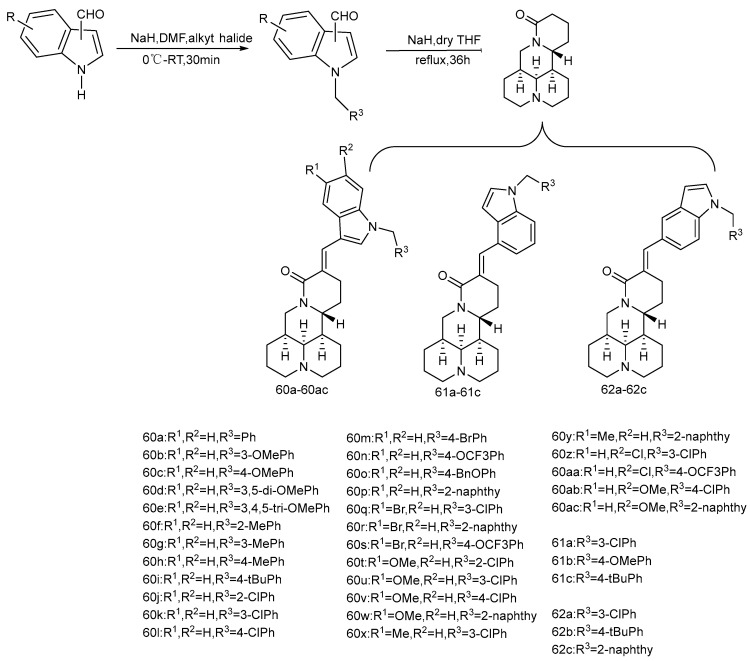
The synthetic route of compounds **60a**–**60ac**, **61a**–**61c**, **62a**–**62c** [68].

The introduction of double bonds and aromatic rings to matrine C-14 can greatly improve its anti-cancer activity, such as YF18 [67] and YYJ18 mentioned above. In addition, the introduction of nitrogen-containing heterocyclic rings is a common strategy for the structural modification of natural products, because nitrogen atoms can affect the interaction between molecules and their targets. Both N-substituted pyrrole group and N-substituted indole group can increase the pharmacological activity of anti-cancer agents. Based on this, Li et al. [68] designed and synthesized a series of 14-(N-substituted-2-pyrromethylene) and 14-(N-substituted—indolemethylene) matrine derivatives (Scheme 7 and Scheme 8). Three human cancer cell lines, SMMC-7721, A549, and CNE2, were selected for in vitro cytotoxicity test. The results showed that all matrine derivatives were more cytotoxic to all three types of cells than matrine. Compounds **59F** and **60u** have the most significant anti-tumor activity, which are superior to the positive control drug cisplatin. Replacing 14-H of matrine with a 1-methyl-2-pyrrolemethylene group offered compound **59A**, which showed poor activity (IC_50_ > 100 μM), while compound **59B** possessing a 1-benzyl-2-pyrrolemethylene group at the same position exhibited a greatly improved anti-cancer activity and was about 115–172 times lower than that of matrine. It could be inferred that the introduction of a benzyl moiety on the nitrogen atom of pyrrole could significantly improve the anti-cancer activity indicating that benzyl might interact with a large pocket within the binding site. The mechanism of anti-proliferative activity of **59F** and **60u** showed that they induced apoptosis of CNE2 and SMMC-7721 cells in a dose-dependent manner, compound **59F** can arrest the cell cycle of CNE2 and SMMC cells in G2/M phase, and compound **60u** does not show significant cell cycle arrest.

**Scheme 9:**
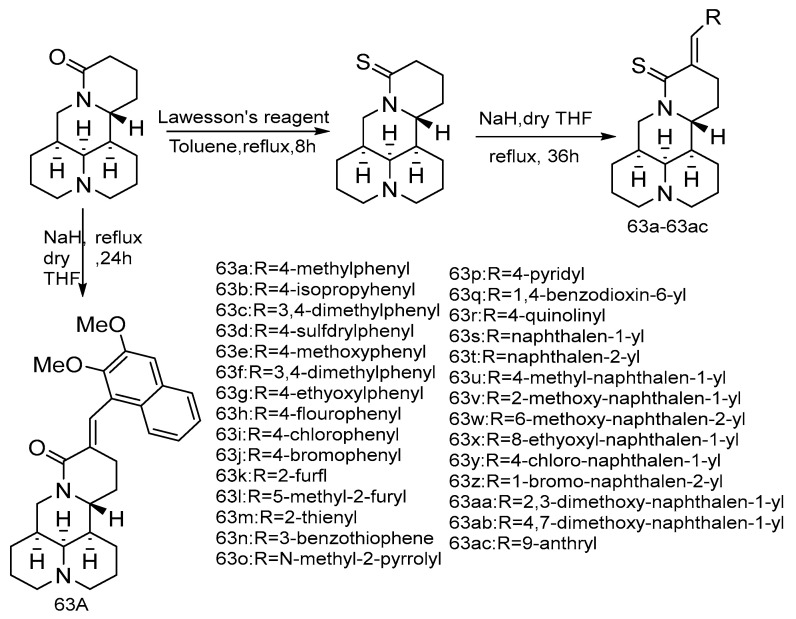
The synthetic route of compounds **63a**–**63ac** [69].

In the same year, the team reported [69] that a series of new thiomarine derivatives were designed and synthesized (Scheme 9) based on the fact that the thiamide group can enhance anti-cancer activity and that the S atom is the bioisostere of the O atom. The results of in vitro cytotoxicity assays of all compounds against human cancer cells A549 and HepG2 show that the derivative **63aa** had the strongest inhibitory activity. Structure–activity relationship analysis shows none of the thiomatrine derivatives bearing furyl, thienyl, pyridyl, quinolyl, and anthryl exhibited inhibitory activity against the two cell lines with inhibition rates over than 50%, a substituted phenylmethylene and naphthalenylmethylene group at C-14 position of thiomatrine was more efficient for the antitumor activity. Notably, among the ten newly synthesized derivatives with a substituted phenylmethylene group on C-14, with a methyl and methoxy group at position-4, the resulting compounds **63a** and **63e,** showed significantly increased antiproliferative activity. The halophenyl derivatives **63h**–**63j** showed weak antiproliferative activities. However, compound **63aa** decorated with 3,5-dimethoxy units on naphthyl showed the most potent inhibitory effect. Removal of methoxy group of **63aa** as shown in **63s** largely decreased the activity and the 14-(9-anthracenemethylene) thiomatrine 63ac did not improve the activity. This result indicates that the addition of electron donating groups on phenyl or naphthalene ring should be favorable for the activity. Changing the position of the dimethoxyl group of compound **63aa** from C-2, 3 to C-4, 7 of the naphthalene ring conferred decreased cytotoxicity on isomer **63ab**. Moreover, the isostere of **63aa** showed a weak inhibitory effect, which proved that the sulfur atom had a good contribution to the inhibitory effect. Further investigation of the mechanism of **63aa** shows that it could induce cell apoptosis by activating caspase-3 and reducing the ratio of Bcl-2/Bax. Compound **63aa** has the potential to be developed as a new anti-cancer drug.

**Scheme 10:**
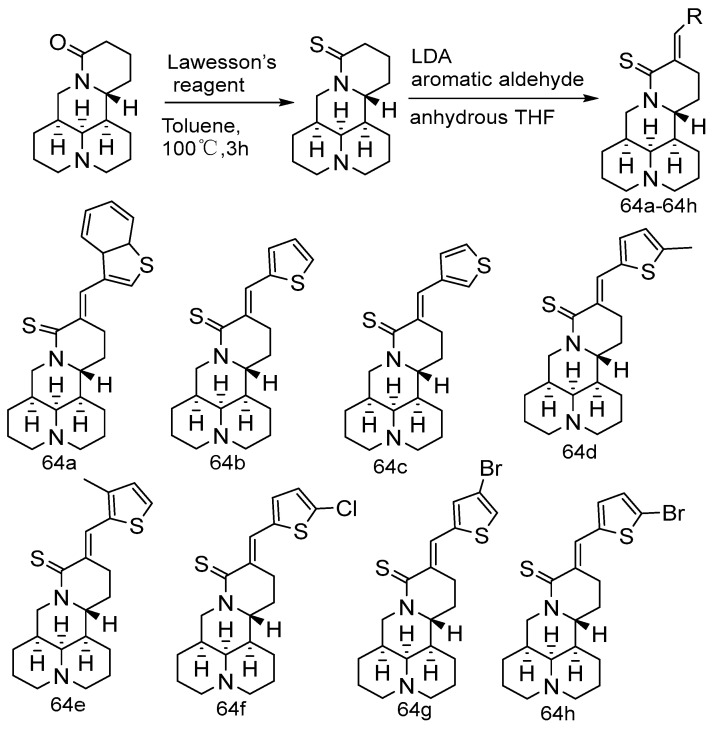
The synthetic route of compounds **64a**–**64h** [70].

It has been reported that matrine can reverse the drug resistance of breast, lung, and bladder cancers. Based on the good cytotoxicity of thiophene compounds in anti-cancer drugs, Wei et al. [70] designed and synthesized eight matrine derivatives (Scheme 10) containing thiophene groups to search for novel compounds that could exhibit significant reversal effects in vitro and in vivo. In vitro activity experiments show that the eight compounds have cytotoxic and preliminary synergistic effects on nasopharyngeal carcinoma cells and cisplatin-resistant nasopharyngeal carcinoma cells (CNE2/CDDP). Compound **64f** and CDDP (cis-diamminedichloroplatinum) show significant synergistic inhibitory effects on leukemia, breast, and bladder cancer cells in vitro and in vivo, based on the high risk of thiophene compounds. The preliminary in vivo toxicity evaluation of 64f was also carried out. The results show that **64f** has no obvious toxicity in mice under physiological conditions.

**Table 3:** Studies on the activity of matrine derivatives structurally modified on matrine C-14 and C-15 from 2018 to 2022.

Compound	Activity Test	Method	Result	References
YYJ18	Antiproliferation activity of YYJ18 on CNE2/5–8F cell	CCK8 assay Plate cloning experiment	The OD value of nasopharyngeal carcinoma cell proliferation after YYJ18 treatment was significantly lower than that in control group, and showed a time-dose dependence, the number of clone formation in the drug treatment group was significantly lower than that in the control group, and the number of clone formation was negatively correlated with the concentration of YYJ18.	[66]
	Effect of YYJ18 on migration ability of CNE2/5–8F cell	Scratch Assay	With the increase of YYJl8 concentration, the migration distance of nasopharyngeal carcinoma cells decreased significantly and showed a negative correlation of concentration	[66]
	Effect of YYJ18 on invasion ability of CNE2/5–8F cell	Teanswell cell invasion test	The invasion ability of CNE2/5–8F cells treated with YYJ18 was significantly weaker than that of the control group, and with the increase of YYJ18 concentration, the number of transmembrane cells was inhibited, showing a negative correlation of concentration.	[66]
	Effect of YYJ18 on tumor formation in nude mice	Pharmacodynamic experiment	The tumor growth rate of nude mice inoculated with CNE2 cells was significantly lower than that of the control group after intraperitoneal injection of YYJ18	[66]
	Effect of YYJ18 on epithelial mesenchymal transformation of nasopharyngeal carcinoma cells	Western blot	In nasopharyngeal carcinoma CNE2/5–8F cells treated with YYJl8, N-cadherin and Vimentin proteins positively correlated with EMT were down-regulated, while e-cadherin proteins negatively correlated with EMT were up-regulated	[66]
	Effect of YYJ18 on PI3K/AKT signaling pathway in nasopharyngeal carcinoma cells	Western blot	The expression of p-Akt protein AKT, a key protein in the P13K/AKT signaling pathway, was down-regulated in nasopharyngeal carcinoma CNE2/5–8F cells after YYJl8 treatment, while the expression of total AKT protein was not significantly changed	[66]
**59**–**62**	Cytotoxicity of compound **59**–**62** to SMMC-7721, A549 and CNE2 cells	MTT assay	The IC_50_ values of all matrine derivatives against SMMC-7721, A549 and CNE2 cells were lower than that of matrine, among which 59F and 60u showed the strongest activity with IC_50_ values of 4.65 ± 0.23, 8.05 ± 0.56, 3.55 ± 0.18 and 3.95 ± 0.34, 4.96 ± 0.54, 3.42 ± 0.23 μM, respectively	[68]
	Effects of compounds **59F** and **60u** on apoptosis of CNE2 and SMMC-7721 cells	Flow cytometry	The percentage of apoptotic cells in CNE2 increased from 4.93% (control) to 11.96% (5 μM), 34.04% (10 μM) and 50.50% (30 μM) respectively after treatment with different concentrations of compound 59F for 24 h. The percentage of apoptotic cells in SMMC-7721 increased from 2.60% (control) to 19.93% (5 μM), 53.58% (10 μM) and 61.50% (30 μM), respectively, the percentage of apoptotic cells of in CNE2 significantly increased from 5.29% (control) to 17.52% (10 μM), 38.60% (20 μM) and 74.20% (50 μM) respectively after treatment with different concentrations of compound **60u** for 24 h. The percentage of apoptotic cells in SMMC-7721 increased from 2.60% (control) to 39.00% (10 μM), 58.80% (20 μM) and 95.00% (50 μM), respectively. The percentage of G2/M phase in CNE2 cells treated with **59F** gradually increased from 19.8% (control) to 29.5% (5 μM), 89.4% (10 μM) and 81.1% (30 μM), the percentage of G2/M phase in SMMC-7721 cells treated with 59F increased from 18.4% (control) to 24.1% (5 μM), 78.9% (10 μM) to 79.1% (30 μM).	[68]
**63a**–**63ac**	Antiproliferation activity of compound **63**–**64**	MTT assay	The IC_50_ values of compounds **63a**, **63c**, **63d**, **63e**, **63f**, **63o**, **63t**, **63v**, **63w**, **63x**, **63z**, **63aa**, **63ab** against A549 cells are 48.3 ± 3.4, 42.6 ± 2.3, 26.6 ± 2.4, 37.7 ± 2.5, 42.7 ± 4.9, 45.7 ± 3.843.0 ± 3.3, 48.1 ± 4.5, 49.6 ± 3.8, 38.7 ± 2.7, 37.8 ± 1.3, 11.4 ± 1.8, 38.8 ± 1.1 μM, respectively. All others are greater than 100 μM. The IC_50_ values of compounds **63a**, **63d**, **63e**, **63h**, **63j**, **63o**, **63t**, **63v**, **63x**, **63z**, **63aa**,**63ab** against HepG2 cells are 35.4 ± 4.4, 24.6 ± 3.2, 26.1 ± 4.1, 45.6 ± 3.7, 36.3 ± 2.6, 49.7 ± 4.3, 37.5 ± 3.4, 45.8 ± 3.8, 39.2 ± 3.1, 34.6 ± 3.5, 9.1 ± 1.2, 25.1 ± 2.9 μM, respectively. All others are greater than 100 μM.	[69]
**63aa**	Antiproliferation activity of compound **63**–**64** against HepG2, SMMC-7721, A549, and CNE2 cells	MTT assay	The IC_50_ values of compound **3aa** against HepG2, SMMC-7721, A549, and CNE2 cells are 9.1 ± 1.2, 9.0 ± 1.4, 11.4 ± 1.8, 7.8 ± 0.8 μM, respectively. The IC_50_ values of matrine against HepG2, SMMC-7721, A549, and CNE2 cells are 4178 ± 395, 5591 ± 521, 5725 ± 602, 5278 ± 498 μM, respectively. The IC_50_ values of cisplatin against HepG2, SMMC-7721, A549, and CNE2 cells are 8.4 ± 0.5, 6.0 ± 0.4, 8.5 ± 0.5, 3.8 ± 0.2 μM, respectively.	[69]
	Effects of compound **63aa** on cell cycle of CNE2 and SMMC-7721	Flow cytometry	The percentage of G2/M phase in SMMC-7721 gradually increased from 19.3% (control) to 19.0% (10 μM), 20.2% (20 μM) and 26.5% (50 μM). Similarly, the percentage of G2/M phase in CNE2 cells increased from 22.0% (control) to 28.2% (10 μM), 31.0% (20 μM) to 34.4% (50 μM).	[69]
	Effects of compound **63aa** on apoptosis of CNE2 and SMMC-7721	Flow cytometry	The percentage of apoptotic cells in CNE2 significantly increased from 2.24% (control) to 24.54% (10 μM), 31.50% (20 μM) and 49.60% (50 μM) after treatment with different concentrations of compound **63aa** for 24 h. Similarly, the percentage of apoptotic cells in SMMC-7721 increased from 2.60% (control) to 19.93% (10 μM), 53.58% (20 μM) and 61.50% (50 μM).	[69]
	Effects of compound **63aa** on the expression of Bax, Bcl-2 and cleavage of caspase-3	Western Blot	**63aa** significantly increased the relative levels of proapoptotic Bax expression but reduced the levels of antiapoptotic Bcl-2 expression in a dose-dependent manner.	[69]
**64a**–**64h**	Cytotoxicity of compound **64a**–**64h** to three NPC cell lines (CNE2, HONE1, and HK-1) and CDDP resistance cell line (CNE2/CDDP)	MTT assay	8 derivatives displayed better antiproliferative effects (IC_50_ ranging from 35 to 700 µM) than the parent compound matrine (IC_50_ more than 1000 µM). Notably, compound **3f** showed the most prominent proliferation inhibition effects in NPC and NPC/CDDP resistant cells with IC_50_ ranging from 30 to 100 µM	[70]
	Combined Inhibitory Effects Evaluation of 8 Derivatives with CDDP	MTT assay	Only compound **64f** could increase the inhibitory effects of CDDP against CNE2/CDDP cells	[70]
	Synergistic Inhibitory Effect Evaluation of Compound **3f** with CDDP	MTT assay	In CNE2/CDDP cells treated simultaneously with 64f and CDDP, the CI values were less than 1, indicating synergism between 64f and CDDP	[70]
	Effect of compound **64f** combined with CDDP on apoptosis of CNE2/CDDP cells	Apoptosis kit	Combined treatment with CDDP (4 µg/mL) and **3f** (40 µM) increased significant cell apoptosis compared with CDDP (15.53 ± 1.61 vs. 7.58 ± 0.82) or **64f** (15.53 ± 1.61 vs. 9.97 ± 1.36) treatment alone	[70]
	In vivo Anticancer Evaluation of Compound **64f**	BALB/C nude mice	The combined treatment of CDDP with 64f significantly reduced tumor volume and weight compared with CDDP or 64f treatment alone	[70]
**65a**–**65i**	Inhibitory activity of compound **65a–65i** against HepG2 and HeLa	MTT assay	The IC_50_ values of compounds **64b**, **64c**, **64d**, **64e**, **64i** and camptothecin against HepG2 were 96, 97, 11, 51, 86 and 39 μM, respectively. All others are greater than 100 μM. The IC_50_ values of compounds **64b**, **64d**, **64e** and camptothecin against HepG2 were 93, 23, 33 and 12 μM, respectively. All others are greater than 100 μM.	[71]

Note: Data are presented as the mean ± SD of three independent experiments.

### 3.3. Modification at Position C-15

**Scheme 11:**
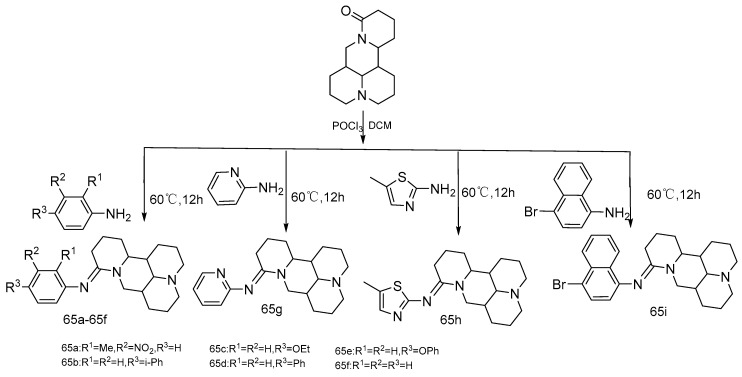
The synthetic route of compounds **65a**–**65i** [71].

There are few reports on the modification of the carbon-oxygen double bond at the 15-position of matrine. Jing et al. [71] synthesized a series of new 15-N-substituted matrine imine derivatives (Compounds **65a**–**65i**) with matrine and 9 arylamines as raw materials, dichloromethane as solvent and phosphorus oxychloride as catalyst (Scheme 11). The IC_50_ values of compounds **65d** and **65e** are less than 50 μM in HepG2 and HeLa cells, suggesting that compounds **65d** and **65e** have potential development prospects.

### 3.4. D Ring Opening and Fusion

It was found that the physical and chemical properties of matrine could be increased by opening the D-ring or merging with other rings. Studies on the pharmacological activities of essential matrine derivatives obtained by ring-opening and fusion modifications of the D ring in the past five years are summarized in Table 4.

**Scheme 12:**
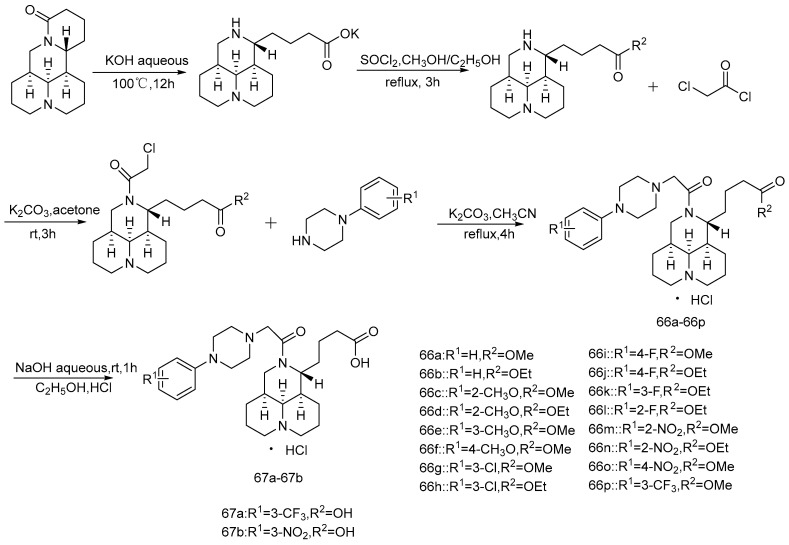
The synthetic route of compounds **66a**–**66p** and **67a**–**67b** [72].

**Scheme 13:**
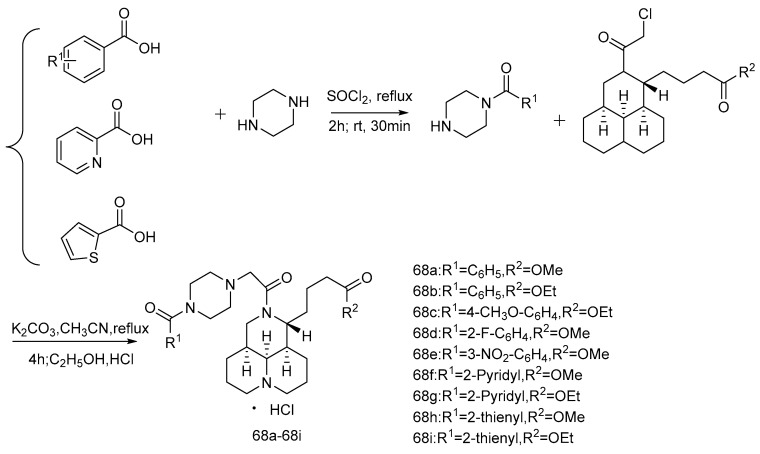
The synthetic route of compounds **68a**–**68i** [72].

In 2019, Xu et al. [72] linked matrine with substituted piperazine derivatives to synthesize 27 target compounds (Scheme 12 and Scheme 13) because of the piperazine structure advantage in drug modification, the antiproliferative activity of all new compounds on human hepatoma cell Bel-7402, and human colon cancer cell RKO and the anti-tumor activity on an H22 tumor-bearing mouse model were evaluated. The results show that the antiproliferative activity of most of the compounds is better than that of matrine. Compounds **66p** and **67a** show strong anti-tumor activity against these two cell lines. The anti-tumor effects of **66a**, **66c**, **66g**, **66j**, **66k,** and **68a** are better than that of matrine in vivo. It can be concluded that the piperazine substituents could enhance the antiproliferative activity and anti-tumor activity of some compounds in vivo.

**Scheme 14:**
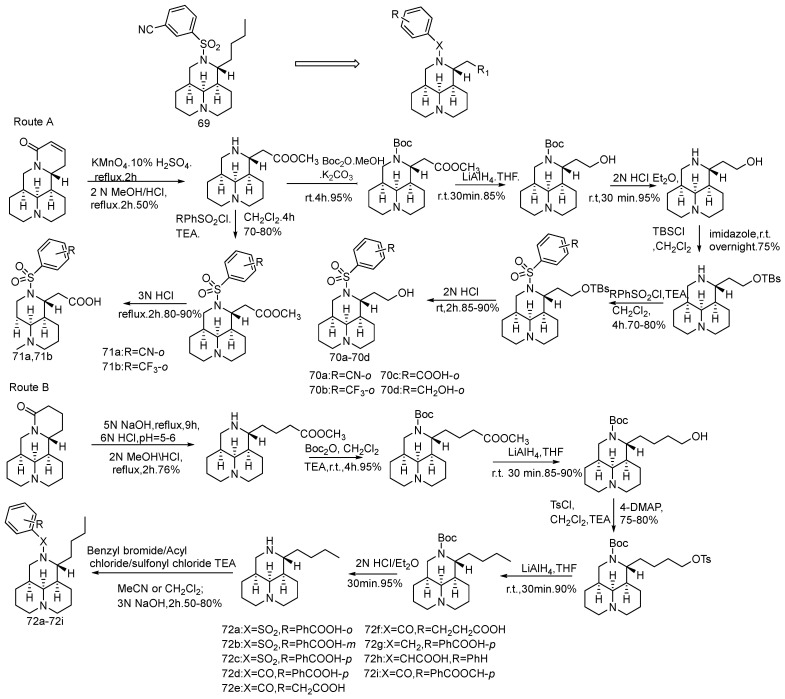
The synthetic route of compounds **70**–**72** [73].

In order to seek new broad-spectrum anti-influenza drugs, based on the identification of 12-N-benzyl matrine derivatives as a class of anti-HBV/HCV virus candidates, Tang et al. [73] screened 12-N-p-cyanophenesulfonyl matrine (compound **69**) against H1N1 influenza virus. Using **69** as the lead, 15 12-N-substituted tricyclic matrine derivatives were designed and synthesized (Scheme 14), and all compounds were evaluated for their anti-H1N1 activity. The results show that **72g** has strong activity.

**Scheme 15:**
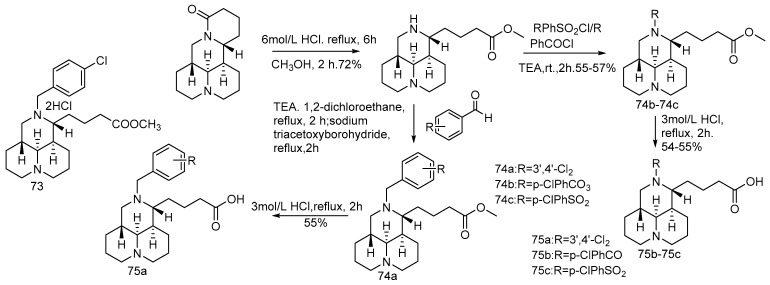
The synthetic route of compounds **74a**–**74c** and **75a**–**75c** [74].

**Scheme 16:**
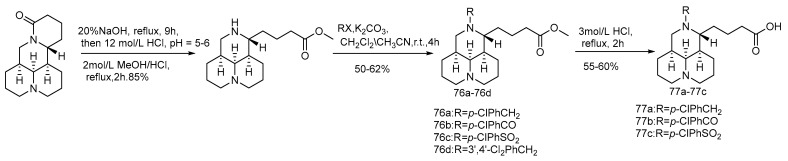
The synthetic route of compounds **76a**–**76d** and **77a**–**77c** [74].

**Scheme 17:**
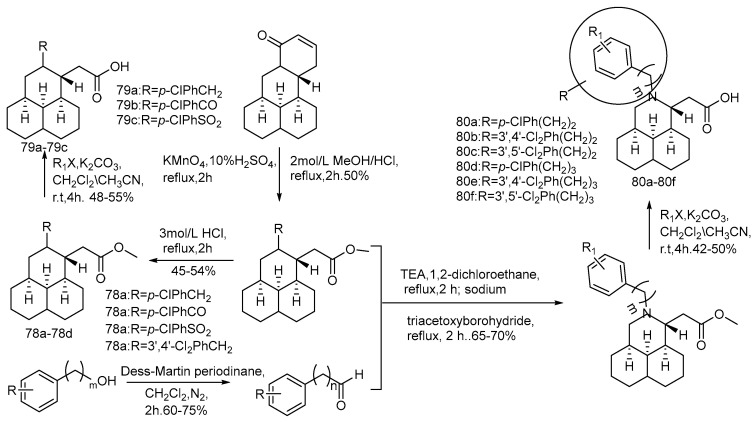
The synthetic route of compounds **78a**–**78d**, **79a**–**79c** and **80a**–**80f** [74].

Professor Song Danqing research team constructed a compound library of tricyclic *Sophorae japonicum* derivatives in the early years. Using sertraline as a positive control, 12-N-p-chlorobenzyl dihydrochloride (compound **73**) shows good anti-EBOV activity in the pseudotype EBOV virus model (pHIV-EBOVGP-Fluc) [74]. Compound **73** is found to have a chlorobenzene fragment at C-12, similar to that of sertraline, suggesting that the chlorobenzene fragment may contribute to the inhibitory effect on EBOV. Based on this strategy, using compound **73** as the lead, 26 novel tricyclic 12-N-substituted sophoracinic acid and oxymatrine acid derivatives were designed and synthesized (Scheme 15, Scheme 16 and Scheme 17), and their anti-EBOV activities in vitro were evaluated. Compound **76d** shows the strongest anti-EBOV activity with an IC_50_ value of 5.29 μM. It is speculated that the 12-N-dichlorobenzene group is beneficial to the improvement of the activity and the chiral configuration of the C-5 may have little effect on the activity.

## 4. Conclusions

**Figure 3:**
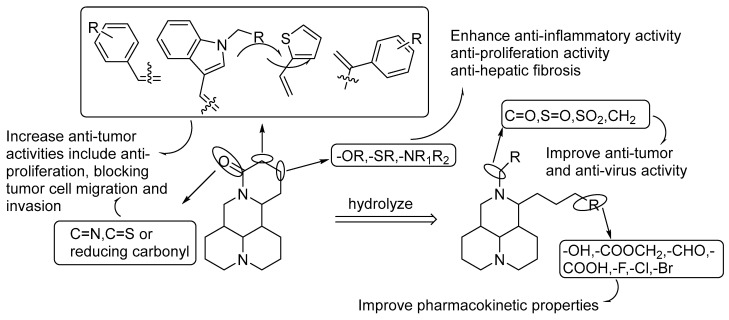
Structure–activity Relationship.

In the past five years, many matrine derivatives have been found and synthesized, and some of their pharmacological activities have been studied. At the end of the article, all the structural modification strategies of matrine and their structure–activity relationships in the past five years are summarized in the form of a diagram (Figure 3). We found that the structural modifications of matrine in these latest studies are still concentrated on the D-ring. However, it is worth noting that the natural compounds with some novel structures obtained from *Sophora flavescens* provide new methods for developing new compounds, such as some matrine-type alkaloid dimers and bisamide alkaloids that have stronger anti-inflammatory activities than that of matrine. Modifying the A and B rings of matrine to obtain new drugs with higher activities and better safety has become a promising and challenging research strategy.

In recent years, there are few reports on the structural modifications of matrine for immunosuppression, cardiac protection, and neuroprotection. In the future, more attention may be paid to finding new modification sites and modification strategies to expand the pharmacological activities and application range of matrine and its derivatives. Hopefully, this review has useful reference value in the subsequent research and development of matrine derivatives.

## 5. Challenges and Prospects

The structure of natural products has complexity and diversity. Traditional research methods (extraction, separation, purification, and identification) are time-consuming and laborious, and most of the components obtained by traditional methods are high content, with stable structure, and it is not easy to obtain trace components or components easily lost in the separation process. In other words, traditional methods cannot achieve rapid identification and efficient identification of unknown components in a large number of samples, which cannot meet the current rapid development of new drug research and development processes. In recent years, there have been a lot of developments in analytical methods (e.g., spectroscopy) for the identification of natural compounds as well as pollutants that can be present in fruits or vegetables [75,76]. There is a large potential in this area of research, especially metabolomics. The emerging discipline of metabolomics has become an important tool in the study of natural products. Metabolomics’ technology aims to reflect the changes of metabolites after stimulation (such as gene mutation, pathophysiological changes, and environmental changes) by conducting large-scale qualitative and quantitative studies on small molecule metabolites of biological systems, therefore, it is suitable for high-throughput, rapid, and comprehensive analysis of natural products. Metabolomics technology has been applied in a wide range of fields, such as disease diagnosis, medical research and development, nutritional food science, toxicology, environmental science [77], botany, and other fields closely related to human health care. The most commonly used analysis techniques of metabolomics are nuclear magnetic resonance (NMR) and mass spectrometry (MS). NMR technology has fast analysis, good selectivity [78], little demand for samples, almost no need for sample pre-treatment, and can provide a wealth of relevant information, such as the structure, concentration, molecular dynamics, and interactions of metabolites [79]. The NMR spectroscopic technique enables us to perform more detailed studies on the issues related to new potentially active or potentially toxic compounds in different types of bio-materials such as animal or plant tissue at the molecular level [80]. MS is often connected in series with different chromatographic systems, offering greater sensitivity and resolution and a wider range of applications than NMR. Metabolomics studies based on MS play an important role in the analysis of natural products, which can obtain a large number of complex and diversified data, such as the precise mass number, elemental composition, characteristic ion fragments, and retention time of compounds.

Matrine is a natural product. It will be of great value to study the druggability and activity mechanism of the modified matrine derivatives and the newly discovered matrine alkaloids by metabolomics. It is worth noting that there are few studies on matrine toxicity at present, and metabolomics can be used as an effective tool to study its toxicity. More importantly, metabolic profiling has the potential to identify toxicity early in the drug discovery process, which could save time and money for the development of new drugs.

## Figures and Tables

**Table 1:** Study on the activity of newly discovered matrine alkaloids from 2018 to 2022.

Compound	Activity Test	Result	References
**2–9**	Inhibitory activity against HBsAg and HBeAg secretion	Compounds **2**, **5**, and **6** had higher inhibition rates on HBsAg and HBeAg secretion than matrine	[43]
**10–14**	Anti-inflammatory Activities in LPS (lipopolysaccharide)-Stimulated RAW 264.7 Cells	The amount of IL-6 produced by the cells treated with the compounds **10**–**13** and matrine was (67.85 ± 0.44)%, (52.87 ± 3.22)%, (73.90 ± 0.34)% (60.08 ± 1.66)% and (65.49 ± 3.64)% of that in the model group; The amount of TNF-α produced by the cells treated with the compound **10**–**14** and matrine was (96.64 ± 1.27) %, (50.05 ± 6.56)%, (49.59 ± 0.51)%, (70.86 ± 0.31)%, (69.14 ± 2.18)% and (72.48 ± 3.83)% of that in the model group	[44]
**15–19**	Immunosuppression experiments	Only compound **18** has a strong inhibitory activity on the proliferation of concanavalin A-induced T lymphocytes and LPS-induced B cells in vitro, with IC_50_ values of 3.98 and 3.74 μM, respectively	[45]
**20–22**	The inhibitory activity on NO production of in RAW 264.7 cells activated by LPS	NG-monomethyl-L-arginine, mono-acetate salt was used as positive control with IC_50_ value of 21.80 μM. Only compound **20** showed good inhibitory effect with IC_50_ value of 18.26 μM	[46]
**23–27**	Anti-inflammatory Activities in LPS-Stimulated RAW 264.7 Cells	The amount of IL-6 produced by the cells treated with the compounds **23**–**27** and matrine was (65.21 ± 0.43)%, (65.89 ± 3.12)%, (73.27 ± 0.28)%, (84.82 ± 1.56)%, (89.26 ± 1.56)% and (82.14 ± 5.75)% of that in the blank group; The amount of TNF-α produced by the cells treated with the compounds **23**–**27** and matrine was (56.82 ± 1.25)%, (85.21 ± 6.21)%, (80.68 ± 0.62)%, (75.86 ± 0.32)%, (66.36 ± 2.24)% and (73.01 ± 1.56)% of that in the model group	[47]
**28–32**	The inhibitory activity on NO production of in RAW 264.7 cells activated by LPS Antitumor activity was tested with Hela, HepG2 and A549 cells; Anti-HIV and anti-influenza virus activities	The IC_50_ value of **31b** was 29.19 μM, and that of matrine was 38.90 μM. The IC_50_ value of other new compounds was greater than 50 μM All compounds showed no anti-tumor activity (IC_50_ > 50 μM) Paclitaxel was a positive control (IC_50_ = 2.54 μM) All compounds showed no anti-HIV and anti-influenza virus activities	[48]
**33–40**	Anti-neuroinflammatory activity in LPS-stimulated BV2 microglia.	All compounds showed anti-neuroinflammatory activity (the medium without LPS were selected as the positive or negative control group)	[49]
**41–44**	Anti-inflammatory Activities in LPS-Stimulated RAW 264.7 Cells	Compounds **43** and **44** can inhibit the release of TNF-α and IL-6 with IC_50_ value from 35.6 to 45.8 μM; Compounds **41** and **42** showed no activity with IC_50_ > 50 μM (Dexamethasone as a positive control group with IC_50_ values of 8.8 ± 1.3 μM and 7.2 ± 0.5 μM for TNF-α and IL-6)	[50]
**45–49**	Anti-inflammatory Activities in LPS-Stimulated RAW 264.7 Cells	The amount of IL-6 produced by the cells treated with the compounds **45**–**49** and matrine was (41.0 ± 1.3)%, (54.7 ± 1.7)%, (85.6 ± 1.4)%, >100%, (84.0 ± 1.6)%, (70.8 ± 2.3)%, (73.3 ± 1.6)%, (71.9 ± 0.9)% and (81.0 ± 1.8)% of that in the model group; The amount of TNF-α produced by the cells treated with the compound **45**–**49** was (34.0 ± 2.9)%, (46.8 ± 1.8)%, (74.7 ± 1.7)%, (90.0 ± 1.5)%, (84.3 ± 1.9)%, (71.7 ± 1.4)%, (73.5 ± 1.0)%, (79.3 ± 2.3)% and (64.3 ± 1.9)% of that in the model group (Dexamethasone as a positive control group with IC_50_ values of 28.8 ± 2.3 μM and 32.2 ± 3.5 μM for TNF-α and IL-6)	[51]
	Cytotoxicity to HepG2, A549, THP-1, and MCF-7 cells	All new compounds showed no obvious cytotoxic activity against those four human cancer cell lines with CC_50_ value more than 100 μmol/L (the positive control, Adriamycin showed inhibition rates that ranged from 75–95%.)	[51]
**50–51**	Anti-proliferative activities against A549 cells.	The IC_50_ value of compound **50** was 20.92 ± 3.03 μM at 24 h, whereas compound **51a** had values greater than 40 μM the IC_50_ values of compound 50 were 12.82 ± 1.39 μM and 7.58 ± 2.47 μM at 48 h and 72 h, respectively	[52]
**50**	The correlation between the anti-proliferative effect and both cell cycle arrest and apoptosis	50-treated A549 cells had increased percentages of early and late apoptotic cells in a dose-dependent manner	[52]
**52–54**	The hepatoprotective effect against APAP-induced hepatotoxicity in HepG2 cells and a mice model	The viability of HepG2 cells induced by APAP increased from 46.21% to 77.50% after treatment with compound **52**; The level of serum Alanine transaminase and aspartate transaminase in model group were 1429.22 ± 82.84 and 1455.47 ± 97.41 U/L, respectively, and in mice administered with compound 52(30mg/kg) were 66.35 ± 11.03 and 169.70 ± 8.67 U/L, respectively	[53]

Note: Data are presented as the mean ± SD of three independent experiments.

**Table 4:** Study on pharmacological activity of matrine derivatives modified by D-ring opening and fusion from 2018 to 2022.

Compound	Activity Test	Method	Result	References
**66**–**68**	Antiproliferative activities of compounds **66**–**68** against human hepatoma Bel-7402 and colorectal carcinoma RKO cells	MTT assay	The survival rates of Bel-7402 cells treated with matrine and compounds **66**–**68** series were (86.6 ± 2.6)%, (78.3 ± 2.1)%, (71.3 ± 3.8)%, (89.4 ± 2.2)%, (85.2 ± 2.4)%, (91.2 ± 3.4)%, (88.3 ± 2.0)%, (71.5 ± 2.5)%, (72.3 ± 3.2)%, (78.4 ± 3.2)%, (90.1 ± 0.2)%, (79.9 ± 0.2)%, (75.9 ± 1.3)%, (84.3 ± 3.5)%, (78.1 ± 4.1)%, (73.3 ± 2.8)%, (50.4 ± 1.4)%, (48.3 ± 2.5)%, (73.5 ± 2.1)%, (81.2 ± 4.8)%, (78.8 ± 2.2)%, (76.1 ± 2.6)%, (81.1 ± 4.3)%, (50.6 ± 2.5)%, (72.2 ± 5.4)%, (73.3 ± 2.3)%, (62.6 ± 3.4)%, (61.3 ± 0.2)%, respectively; The survival rates of RKO cells treated with matrine and compounds **66**–**68** series were (84.3 ± 2.5)%, (81.7 ± 2.1)%, (80.2 ± 2.4)%, (83.8 ± 1.2)%, (75.2 ± 2.6)%, (88.0 ± 1.8)%, (88.7 ± 3.1)%, (76.1 ± 2.5)%, (75.7 ± 2.7)%, (79.8 ± 2.2)%, (79.1 ± 4.6)%, (84.1 ± 3.4%), (72.4 ± 3.8)%, (53.4 ± 1.8)%, (43.8 ± 2.5)%, (58.1 ± 3.7)%, (48.3 ± 3.1)%, (51.3 ± 3.3)%, (76.2 ± 2.7)%, (78.1 ± 2.0)%, (82.6 ± 2.4)%, (87.5 ± 4.8)%, (81.5 ± 2.7)%, (55.2 ± 1.3)%, (76.2 ± 2.7)%, (76.7 ± 3.5)%, (75.8 ± 1.2)%, respectively.	[72]
**69**–**72**	In vitro anti-flu activities against influenza A/Puerto Rico/8/34 (wild H1N1)	Viral cytopathogenic effect assay	The IC_50_ values of compounds **69**–**72** series, Ribavirin and Oseltamivir were 10.7, 19.0, 51.4, 94.2, >169.0, 55.1, 115.9, >158.1, 108.2, >620.4, >594.4, >540.5, >539.8, >501.9, 13.0, 5.3 μM	[73]
**73**–**80**	In vitro anti-EBOV activities of compounds **73**–**80** in human embryonic kidney (HEK) 293 T cells.	--	The IC_50_ values of compounds **73**–**80** series and Sertraline were 5.07, 2.68, 39.1, 2.90, 78.6, >200, 11.4, 25.4, 88.2, 8.23, 5.29, >200, >200, 30.2, 28.1, 88.2, 26.8, 10.0, >200, >200, >200, 83.3, >200, 83.3, >200, 83.3, 83.3, 1.08 μM	[74]
**76d**	Anti-EBOV and anti-MARV activity in vivo of the compound **76d**	BALB/c mice	Compound **76d** displayed anti-EBOV activity by contributing a 65% reduction, significantly higher than that of sertraline (45%). Compound **76d** displayed anti-MARV activity by contributing a 50% reduction, while sertraline displayed no activity at all.	[74]

Note: Data are presented as the mean ± SD of three independent experiments.

## Data Availability

Not applicable.

## Sample Availability

Samples of the compounds are not available from the authors.

## Abbreviations

AMPK	adenosine 5′-monophosphate-activated protein kinase
APAP	acetaminophen
Bax	Bcl-2 Assaciated X protein
Bcl-2	B-cell lymphoma-2
CDDP	cis-diamminedichloroplatinum
COX-2	cyclooxygenase-2
EMT	epithelial-mesenchymal transition
GSK3β	recombinant Glycogen Synthase Kinase 3 Beta
IL-6	interleukin-6
iNOS	inducible Isoform of Nitric Oxide Synthase
LPS	lipopolysaccharide
MTT	3-(4,5)-dimethylthiahiazo(-z-y1)-3,5-di-phenytetrazoliumromide
PI3K	phosphatidylinositol3-kinase
ROS	reactive oxygen species
RPS5	ribosomal protein S5
TGF-β1	transforming growth factor beta 1
TNF-α	tumor necrosis factor-α
